# New Composite Packaging Material from Edible Oil By-Product Coated with Paraffin Wax for Dry Apricot Slice Packing Under a Modified Atmosphere

**DOI:** 10.3390/polym16243583

**Published:** 2024-12-21

**Authors:** Nevena Hromiš, Sanja Ostojić, Lato Pezo, Senka Popović, Zdravko Šumić, Anita Milić, Sunčica Kocić-Tanackov, Danijela Šuput

**Affiliations:** 1 Faculty of Technology Novi Sad, University of Novi Sad, Bulevar cara Lazara 1, 21000 Novi Sad, Serbia; nevena.krkic@uns.ac.rs (N.H.); madjarev@uns.ac.rs (S.P.); sumic@uns.ac.rs (Z.Š.); anitavakula@uns.ac.rs (A.M.); suncicat@uns.ac.rs (S.K.-T.); 2 Institute of General and Physical Chemistry, University of Belgrade, Studentski Trg 12, 11000 Belgrade, Serbia; ostojicsanja404@gmail.com (S.O.); latopezo@yahoo.co.uk (L.P.)

**Keywords:** packaging, biopolymer, hydrogel, pumpkin oil cake, dry apricot slices, modified atmosphere

## Abstract

Composite biopolymer hydrogel as food packaging material, apart from being environmentally favorable, faces high standards set upon food packaging materials. The feature that favors biopolymer film application is their low gas permeability under room conditions and lower relative humidity conditions. However, most biopolymer-based materials show high moisture sensitiveness and limited water vapor permeability, which limits their application for food packaging. In this paper, a new packaging material derived from an edible oil industry byproduct (pumpkin oil cake) coated with a thin layer of paraffin wax was obtained. Compared to the film without wax coating, the new material showed reduced water sensitivity and significantly reduced water vapor transmission rate (56.98 ± 7.42 g/m^2^ 24 h). The new material was tested for packing dry apricot slices under a modified atmosphere (100% N_2_). Gas composition in PuOC/wax pouches’ headspace was minimally changed during 105 days of storage. The low moisture content (6.76–10.60%) of dried apricot slices was preserved throughout the storage period (*p* > 0.05), as well as high rehydration power (65–75%). Changes in sensorial properties during storage were minimal. Total phenol content was minimally reduced during storage, followed by antioxidant activity (FRAP and ABTS trial). The microbial profile of dried apricot slices showed that a safe product was obtained throughout the storage. Considering the results, the functionality of new material for packing dry apricots under a modified atmosphere was proven.

## 1. Introduction

Modern lifestyles increase the demand for healthy, fresh and convenient food, especially foods adapted for “on-the-go” consumption. The growing popularity of healthy snacks with health-conscious consumers is reflected also in the packaging [1,2]. An inevitable retainer of food processing, providing safe and convenient distribution from manufacturer to consumer, is packaging [3,4]. The functions of packaging are numerous, and besides containing products, food packaging must comply with regulations for food contact materials, be convenient, satisfy economic and environmental demand, represent and communicate the product [5]. When dry fruit and snack packaging are analyzed, the most important factor concerning shelf life is the moisture barrier property of the packaging material. Traditionally, a material is considered suitable if it keeps the moisture absorption of the product to less than 3% during the declared shelf life [6]. It should be kept in mind that water content in most fruit snack products is very low and any absorbed moisture would result in the loss of the desired crisp and crunchy feel of the product, leading to the unacceptable sensory textural experience of gummy products [7]. The hygroscopic nature of the product represents an additional challenge. The gain of moisture can also lead to microbial contamination, changing product texture, color, flavor, and nutritional quality [4]. As it was reported that dry fruits and vegetables tolerate low levels of O_2_ in the surroundings (>0.5%), without stress upon living plant tissue, unlike their fresh counterparts (for apricots > 2%), this information could be utilized in low-oxygen modified atmosphere packaging. This packaging could additionally preserve dry fruit or vegetables from oxidative changes of highly valuable nutrients and, at the same time, limit the growth of spoilage microorganisms [8,9]. Different modified atmospheres, usually composed of N_2_ with or without a certain amount of CO_2,_ are mostly used for dry fruits, nuts, and chips. Packing food in gas barrier packaging materials using an inert gas atmosphere like N_2_ can provide a shelf life of 12 months for snack products and dry fruits, provided that the amount of light in the packaging, as an oxidation catalyzer, is minimized [8]. Ensuring a low level of O_2_ inside packaging during a prolonged shelf life includes multiple challenges, with some of them being barrier material usage, heat seal quality, packaging machine setting, and product handling during distribution and storage. Apart from postponing the oxidative reaction, using a N_2_-modified atmosphere creates a “cushion” that protects fragile snack food’s mechanical integrity during storage and manipulation [1,5,10].

A common design for fruit and vegetable snack packaging, as well as for dry fruit, that is currently present on the market is flexible packaging, amounting to almost 80% [6]. There are many packaging material compositions that have proven their efficiency in chip packaging, with the most common being oriented polypropylene (BOPP), polyester (PET) and polyethylene (HDPE or LDPE), alone or in combinations with additional metallization or an aluminum layer for higher barrier properties. Some of the typical structures are BOPP/LDPE, BOPP/PET/LDPE, PETmet/LDPE, BOPP/PETmet/LDPE, PET/LDPE and PET/Al/LDPE. Also, the outer layer is often thermoplastic resin that can add strength and puncture resistance to the bag [11,12].

The environmental aspect of packaging materials has been obtaining increasing attention in the packaging industry due to the new legislation, as well as increasing consumer environmental awareness [5,13]. Some of the examples of environmentally friendly chip packaging designs that can be found on the market are multilayer high-barrier recyclable packaging excluding metalized and aluminum layers, packaging based on compostable PLA or coated cellulose films with improved moisture barrier properties [14,15].

A by-product of cold-pressing the oil out of pumpkin seeds (*Cucurbita pepo* L.) is pumpkin oil cake (PuOC). It contains 63% proteins, 12% carbohydrates, 8.4% oils, 4.5% crude fibers, and 13% other components [16]. PuOC was successfully employed to produce strong and elastic edible cast films with good gas barrier properties, which could be a new method of PuOC utilization [17]. However, film water vapor sensitivity and permeability remain an issue to be dealt with [18].

With the aim to pack dried apricot slices (apricot chips) in a modified atmosphere, a novel composite biopolymer hydrogel-based film [19] composed of a PuOC layer and a paraffin wax layer was investigated. To reduce the water sensitivity and water vapor permeability of PuOC films, a coating of paraffin wax was applied. After being vacuum-dried, the fresh apricot was packed in novel two-layer material and kept for 3.5 months at room conditions. To verify the new packaging system’s appropriateness for packing dry fruit and vegetable products in a modified atmosphere, tests were conducted on the packaging quality, microbiological profile, and apricot technological parameters of quality during storage.

## 2. Materials and Methods

### 2.1. Packaging Material and Packaging

Pressed hull-less pumpkin (*Cucurbita pepo* L. *cv. Olinka*) oil cake (PuOC) was generously provided by “Linum” (Čonoplja, Serbia). “Tipoplastika” (Gornji Milanovac, Serbia) generously provided the 47 µm thick packing material, printed duplex of oriented polypropylene with metalized polypropylene (OPPmat/B/adh/OPPmet) and wax “Paraflex” (Heerhugowaard, The Netherlands). Sigma-Aldrich Chemical Co. (St. Louis, MO, USA) provided the zein. All other applied reagents were of analytical grade.

### 2.2. Packaging Material Production and Pouch Formation

Initially, a PuOC film-forming suspension (FFS) was cast to create the PuOC composite biopolymer hydrogel-based layer. The 10% *w*/*w* PuOC was suspended in deionized water with 30% *w*/*w* glycerol (g/g PuOC). After adjusting the pH of the resulting FFS to 12.0 using 0.2 M NaOH, the FFS was incubated for 20 min at 90 °C. After passing through a nylon filter, hot FFS was poured onto a Teflon-coated flat glass surface [17]. The cast mass of FFS was 0.143 g/cm^2^. Film samples were allowed to air dry at room temperature for two days.

In the second step, melted “Paraflex” wax was coated on the surface of PuOC composite film at 0.003 g/cm^2^. The wax was melted in an oven (Sterimatic ST-11, Instrumentaria, Zagreb, Croatia) at 250 °C and coated on PuOC. Two-layer material was cut into pouch sides 120 mm × 150 mm.

**Figure 1:**
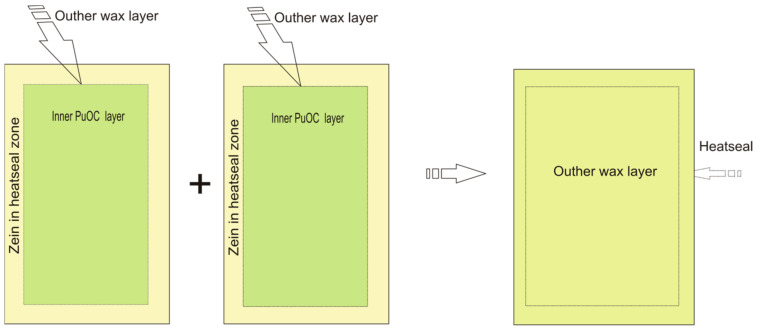
PuOC pouches’ formation steps: two material sides with inner PuOC layer (green), outer wax layer (khaki) and zein in the heat seal zone (beige) overlap to be heat-sealed and form a pouch.

In the third step, zein solution was prepared by suspending zein at 10% *w*/*v* in 85% ethanol as well as PEG 400 per weight of zein (50% *w*/*w*). This solution was applied using a brush in the heat seal zone of the inner side of pouches (Figure 1). The role of zein was to enable pouch formation by forming a heat seal. It had no contribution to other packaging properties (mechanical, barrier, etc.).

A laboratory packing machine (Audion Elektro, Swissvac, The Netherlands) was used to produce the pouches. After three heat seals were formed, 20 g of dried apricot slices was placed in each pouch and the pouches were sealed using the packer BOXER 35 (HENKELMAN vacuum systems, ‘s-Hertogenbosch, The Netherlands), with the fixed gas composition of 100% N_2_ (Gourmet N, Messer Tehnogas, Novi Sad; WITT pressure receiver, WITT-GASETECHNIK GmbH & co KG, Witten, Germany). A modified atmosphere consisting of 100% N_2_ was chosen following previous experiments where tests showed that PuOC-based materials cannot maintain high CO_2_ levels for a prolonged storage period. To compare new packaging with the packaging solution already in use for similar applications, the same conditions were applied to packed apricot chips in OPPmat/B/adh/OPPmet pouches of the same dimensions, formed and closed using the same packing machine. Closed pouches with apricot chips were stored under room conditions for 3.5 months and different properties were analyzed on the 0, 15th, 45th and 105th day of storage.

### 2.3. Properties of Packaging Material and Packaging

Film thickness was gauged with a micrometer Digico 1 (0.001 mm sensitivity) (Tesa, Renens, Switzerland). The measurement was performed in eight replicates for each sample [18].

For water content determination, composite biopolymer hydrogel-based film samples (2 × 2) cm were weighed (w_1_) and dried at 105 °C until a constant mass was reached (w_2_). Water content (WC) was given on a wet basis and calculated as the proportion of initial film weight lost after drying (Equation (1)).

WC [%] = ((w_1_ − w_2_) × 100)/w_1_ (1)

Weighed in air-dried conditions (w_1_), the pieces of composite biopolymer hydro-gel-based films (1 × 2) cm in size were used to measure swelling ability. After that, they spent two minutes submerged in deionized water at 25 °C. After wiping wet samples with filter paper to remove extra liquid, they were weighed once more (w_2_). Equation (2) was used to calculate the amount of absorbed water.

Swelling ability [%] = ((w_2_ − w_1_) × 100)/w_1_ (2)

where w_2_ and w_1_ are the weights of the wet and the air-dried samples, respectively [20].

For the determination of total soluble matter (TSM), small squares of composite biopolymer hydrogel-based films measuring 2 × 2 cm were dried in an oven at 105 °C until a constant weight was achieved. After drying, the film samples were placed in glass containers with 20 mL of deionized water. The glass containers closed, gently agitated, and left for 24 h at 23 ± 2 °C. Finally, water was decanted and film samples were dried at 105 °C until a constant weight was reached. The TSM of the films was calculated using Equation (3).

TSM [%] = ((w_1_ − w_2_) × 100)/w_1_ (3)

where w_1_ and w_2_ are the dry masses before and after the test, respectively [17].

Tensile strength (TS) and elongation at break (EB) were evaluated using the 4301 Instron Universal Testing Machine (Instron Engineering Corp., Canton, MA, USA), following the ASTM standard D882-10 [21]. A strip of film measuring 90 mm by 15 mm was utilized for the tests. The initial separation of the grips was established at 50 mm, with a crosshead speed of 50 mm per minute. Measurements for TS and EB were conducted on a minimum of five samples for consistency.

The water vapor barrier properties were assessed through a gravimetric method (dish method), following the guidelines of ISO 2528:1995 [22] under condition A (with a temperature of 25 ± 1 °C and relative humidity of 90 ± 2%, achieved using a saturated potassium nitrate solution). Silica gel served as a desiccant in the testing apparatus.

The composition of the atmosphere within pouches was analyzed using OXYBABY (WITT-GASETECHNIK GmbH & co KG, Germany). Evaluations were carried out immediately after pouch formation and subsequently at intervals of 15, 45, and 105 days of storage, with results shown as the average of three replicates.

To identify seal leaks in pouches, a dye penetration test was conducted using a solution of 0.5% rhodamine in ethylene glycol monoethyl ether, following ASTM F1929-15, Method A [23]. Any pouches found to have seal leaks were discarded.

### 2.4. Apricot Drying and Characterization

Raw apricots (*P. armeniaca*) were acquired from the market in Novi Sad, Serbia. Each apricot fruit was first cut in halves and then each half was sliced into 5–6 slices, with a thickness of 2–3 mm. To prevent potential deterioration, samples were frozen after slicing at −20 °C until drying was carried out.

The vacuum drying process was described in detail by [24]. The vacuum drying conditions were the following: 55 °C, 20 mbar and 4 h 30 min.

For dried apricot analyses, methanol extract was prepared. Dried apricot samples were processed using a blender, and approximately 5.0 g from each ground sample was covered with 50 mL of methanol, which served as the extraction solvent. Following a 24-h extraction period, the resultant extracts were filtered, transferred into glass containers, and kept in the refrigerator to avoid oxidation until further analysis. The methanol extracts were utilized for determining total phenolic and total flavonoid content, and assessing antioxidant activity through DPPH, FRAP, and ABTS assays.

The analysis of the parameters including moisture content (MC%), water activity (a_w_), and rehydration power (RP%) was carried out according to the method presented by Tepić Horecki et al. [25]. In brief, moisture content was determined using gravimetric analysis by drying the sample at 105 ± 0.5 °C until a constant mass was achieved [26]. The moisture content was calculated by subtracting the percentage of dry matter content from 100%. The water activity (aw value) was determined by placing approximately 2.5 g of the ground sample into a plastic measuring cup of the measurement cell with a sensor inside the water activity meter (LabSwift, Novasina, Switzerland). After equilibrium humidity was reached, the aw value was read from the display of the hwater activity meter. Rehydration power was determined by measuring the volume of water not absorbed by the dried samples [27]. Dried samples were ground, and 2 g of the sample was transferred into a 100 mL measuring vessel, which was then filled with 50 mL of distilled water. The vessel was covered with aluminum foil and left in a dark place for 24 h. After 24 h, the content was filtered, and the volume of unabsorbed water was measured.

The total phenolic content (TP) (mg GAE/100 g) was assessed using the Folin–Ciocalteu method [28], while the total flavonoid content (TF) (mg CE/100 g) was determined through a chloride colorimetric assay [29]. The results were expressed as mg of gallic acid equivalent and mg of catechin equivalents (CEs) per 100 g of dry weight, respectively.

The capability of the sample to reduce Fe^3+^ ions, known as FRAP, was evaluated by a slightly modified procedure initially developed by Benzie and Strain [30]. Absorbance was recorded at 593 nm using a UV-VIS spectrophotometer (6300 Spectrophotometer, Jenway, Dunmow Essex, UK) in triplicate. Calibration was achieved with freshly prepared aqueous solutions of Fe^2+^ (Fe_2_SO_4_) ranging from 0 to 0.23 mM (R² value of 0.999). The results were reported as milligrams of Fe^2+^ equivalents per gram of dried apricots (mg Fe^2+^/g).

The scavenging activity against 2,2-diphenyl-1-picrylhydrazyl (DPPH∙) free radicals was assessed using a modified approach based on the method by Brand-Williams, Cuvelier, and Berset [31], using a VIS spectrophotometer (6300 Spectrophotometer, Jenway, Dunmow Essex, UK). The calibration curve was created from measurements of free radical scavenging using freshly prepared aqueous solutions of Trolox ranging from 0 to 0.8 mM (R² value of 0.984). The results were expressed as milligrams of Trolox equivalents per gram of dried apricots (mg TE/g).

The sample’s ability to scavenge ABTS free radicals was determined through an adapted method initially described by Re et al. [32]. Absorbance readings were taken at 734 nm in triplicate using the UV-VIS spectrophotometer (6300 Spectrophotometer, Jenway, Dunmow Essex, UK). The calibration curve was established with freshly made Trolox aqueous solutions ranging from 0 to 0.8 mM (R² value of 0.987). The results were reported as milligrams of Trolox equivalents per gram of dried apricots (mg TE/g).

All experiments were conducted in triplicate, with results reported as mean values with standard deviations indicated.

The sensory analysis was performed by a seven-member panel of employees of the Faculty of Technology Novi Sad, University of Novi Sad, with previous experience in testing dried fruit. The testing was conducted on the first (0) day and then after 15, 45 and 105 days of dried sample storage in two different tested packaging systems [33]. Panelists were asked to rate samples by using a scale (worst to best) for each feature to be evaluated. Accordingly, the scale of 0 (worst) to 6 (best) was used for testing color properties and general state, including appearance, and likeability, while the scale of 0 (worst) to 4 (best) was used to test smell and taste of dried samples. The overall score was 20 in total.

Sample preparation for microbiology analysis was performed under aseptic conditions on the first (0) day and then after 15, 45 and 105 days of dried sample storage. A total of 20 g of chopped dried apricots was blended with 180 mL of sterile Buffered Peptone Water (Merck KGaA, Darmstadt, Germany) and agitated for 15 min at a speed of 200 rpm using a Unimax 1010 shaker (Heidolph, Schwabach, Germany). Decimal dilutions were created up to a factor of 10^−3^. From each dilution, 1 mL was transferred to sterilized Petri dishes, followed by the addition of suitable media dependent on the specific microorganisms being tested. The microbiological assessments included total aerobic mesophilic bacteria count [34], total yeast and mold count [35] and total Enterobacteriaceae count [36], as well as testing for *Salmonella* spp. [37], Escherichia coli [38], and Listeria monocytogenes [39]. The results were reported as CFU/g.

Statistical evaluations were performed with OriginPro 8 software (OriginLab Corporation, Northampton, MA, USA) [40]. Each measurement was conducted in at least three replicates for each sample, and all results were presented as mean values along with their standard deviations (mean ± SD). ANOVA was utilized for variance analysis, maintaining a confidence level of 95% (*p* < 0.05), and the Tukey test was applied for comparing means.

## 3. Results

The properties of two new packaging materials used for dried apricot slice packaging in a modified atmosphere are given in Table 1. For the illustration, literature data for the PuOC film are also shown.

**Figure 2:**
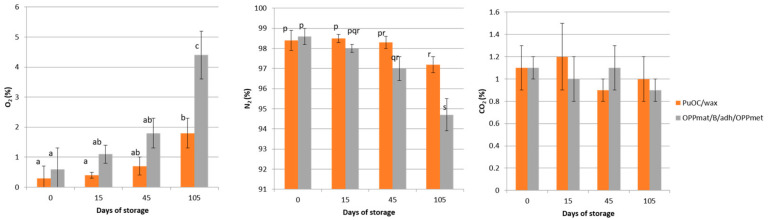
Headspace gas composition (%) in PuOC and OPPmat/B/adh/OPPmet-based pouches during 105 days of storage. ^a,b,c^ Different letters denote significantly different means for O_2_ concentration with 95% probability (*p* < 0.05); ^p,q,r,s^. Different letters denote significantly different means for N_2_ concentration with 95% probability (*p* < 0.05).

As can be seen from Table 1, the new packaging material PuOC/wax inherited most of the tested properties from the PuOC layer, showing relatively low tensile strength with satisfactory elongation at break, taking into account biopolymer-based films [41,42]. The tensile strength of the PuOC/wax film was lower than the average TS values of biopolymer polysaccharide-based films, while the EB value was higher than the majority of these films (chitosan, starch, agar, pectin, pullulan). When compared with protein biopolymer films (zein, composite films based on gelatin, whey protein and collagen), a similar relation was observed. For the aliphatic polyester biopolymers (PLA, PBAT, PCL and PHBV), PuOC/wax is ahead of most when it comes to EB, while TS is comparable [43]. The presented results for swelling degree and total soluble matter demonstrate the composite biopolymer hydrogel-based film’s water sensitivity, which was also derived from the PuOC layer, although the moisture content was considerably lower in the PuOC/wax film, compared to PuOC film. These values are in the range characteristic of hydrophilic, water-sensitive biopolymer films [41]. The most significant alteration in the PuOC film properties was noticed for the water vapor transmission rate value, where laminating the PuOC layer with a wax layer led to a water vapor transmission rate value that was 6 times lower compared to the plain PuOC film. This value is still not competitive compared to conventional polyethylenes, but it is a major improvement for biopolymer-based material [41,44]. Compared with other biopolymer-based materials, such as methylcellulose, whey protein isolate, chitosan, wheat and quinoa starch, as well as many residual fruit and vegetable flour-based materials, the same improvement was shown [45]. If this property is evaluated with the gas barrier properties of PuOC film under room conditions, 12.1 mL/m^2^/day at 1 bar O_2_, as published in earlier research [17], produced two-layer material could be considered as a possible solution for packing sensitive valuable food products with reduced moisture content, like dry fruits, under modified atmosphere conditions. Pouches were successfully formed and the absence of heat-seal leaks was confirmed. Pouches were filled and sealed under a modified atmosphere (100% N_2_). In Figure 2, the composition of the modified atmosphere in tested pouches during storage is given.

The results presented in Figure 2 indicate that the dried apricot slice packaging process was performed with a well-controlled modified atmosphere composition in closed pouches (0 days of storage), while during storage, the atmosphere composition changed in both the analyzed pouches (*p* < 0.05). This is probably because of low respiration rates in dried fruit (<1 mg CO_2_/kg h at 5 °C), as opposed to fresh and minimally processed fruits, mainly because of the lower water content and the conditions of the tissues [46]. No increase in CO_2_ was recorded (*p* < 0.05), which is in accordance with a low respiration rate. The stable level of CO_2_ in the packages was followed by a slow but constant increase in O_2_ concentration, probably due to material permeation and heat seal micropores. This minimally expressed increase in oxygen concentration became statistically significant after 105 days of storage. During storage, atmosphere changes were less pronounced in PuOC/wax pouches, compared to commercial packaging material OPPmat/B/adh/OPPmet pouches (*p* < 0.05). This difference between the two packaging systems was captured only after 105 days of storage. Still, it could be said that both pouches gave satisfactory results as MA packaging systems for dried apricot slice storage for 3.5 months under room conditions. These results are in support of the hypothesis that PuOC/wax-based pouches can be successfully used for packing under a modified atmosphere. It is very interesting to notice that modified atmosphere stability over 3.5 months in PuOC/wax pouches can be compared to high-barrier material with a metalized layer (in this study), as well as with PET/Al/PE used for dry apricot slice packaging [12,47] and PET/Al/BOPA/LDPE used for dry mango slice packing under MAP [48]. PuOC/wax pouches preserved MAP composition better than some other synthetic polymer-based materials used for dry apricot packaging, such as PA20/PP75, PET/PE, PAP/PE and PAP/Al/PE [12,46]. Changes in the oxygen level in commercial packaging in this paper are in agreement with literature data for similar packaging material, such as BOPP/metBOPP used for packing sweet potato chips in 100% N_2_ [11]. The value of this result is particularly important in the context of renewable green materials and the circular economy approach.

The physico-chemical properties of fresh apricot were as follows: 89.74 ± 1.30% moisture content, a_w_ value of 0.966 ± 0.002, 1.51 ± 0.07% proteins, 0.88 ± 0.02% cellulose, 1.22% acidity and 41.37 ± 0.42 mg GAE/100 g content of total phenolic compounds, total flavonoid content of 5.27 ± 0.37 mg CE/100 g, and total sensory score of 20 ± 0. The quality parameters of dried apricot slices before packaging and during storage are reported in Table 2.

After drying, the moisture content in apricot slices was lowered from the initial 89.74 ± 1.30% to 11.44 ± 0.52%. This value follows national regulations for dry fruit, where the moisture content of dry fruit should be below 27% [49]. Compared with similar products, moisture content after drying is similar to the moisture content in dry apple chips (11–20% after drying), dry papaya pieces (13.5%), and microwave-convective dried apricot samples (14.48–23.57%) [50,51,52], which is considerably lower than in the dry apricot samples reported by anđelovicetal. [47] and Ranđelović et al. [12] (31.2%) and Miranda et al. [7] (27.14%). In dry fruit products during storage in room conditions, the main risk was identified as a moisture content and water activity increase. During 105-day storage in MAP, there was no notable difference in the moisture content of apricots, compared to initial moisture content, in both analyzed packaging systems, which is an important proof of the functionality of new PuOC-wax packaging. These results follow similar research where dry fruit was stored under MA at room conditions using high-barrier materials [7,11,47,48,53], while in other reported studies, moisture content increased, although high-barrier materials were used for packaging [40,47].

Preserving low moisture content during storage certainly contributed to high sensory scores throughout the storage. The total sensory score (TSS) of a maximum of 20 at the beginning of storage was slowly reduced during storage, without a significant difference between the two packaging systems tested. Final sensory scores of around 19 for both samples packed in PuOC/wax and OPPmat/OPPmet pouches can be considered as high sensory scores. Changes in sensorial properties during 105 days of storage were minimal.

Drying apricots as a raw material with suitable nutritional and sensorial characteristics has been the subject of research carried out by many authors [54,55,56]. It was reported that the drying process, ascribed to the heat applied, might lead to certain physico-chemical changes in fruit constituents, such as reduction in the starch hydration degree, reduction in the flexibility of cell walls and coagulation of proteins, resulting in their lower water-holding capacity. These changes affect the rehydration power of dry fruit and the rate and extent of rehydration can serve as a measure of food quality, as well as a measure of the extent of changes made to the fruit material through drying and pre-drying processes [25,57,58]. As presented in Table 2, the rehydration power of dried apricot slices was very high at the beginning of storage. Tested samples were able to absorb up to 70% of the initial water content. This result indicates the high quality of the obtained product. During storage, a minimal decrease in RP was noticed for samples packed in OPPmat/OPPmet pouches (*p* < 0.05), while samples packed in the PuOC/wax pouches preserved the initial RP of packed samples throughout the storage period.

Plant secondary metabolites, among which flavonoids and phenolic compounds occupy a noteworthy place, have been exhaustively investigated for their possible effects on the lower frequency of cancers and chronic diseases, such as cardiovascular diseases and type II diabetes. Longer-term modest intake of these compounds was related to beneficial health effects [59,60].

During storage in both packaging systems tested, total phenol content decreased (*p* < 0.05). A similar trend of decrease in total phenol content was also reported for dry papaya samples stored in PET/Al/PE and PA/PE barrier packaging materials under atmospheric conditions and 30 ± 2 °C, dry apricot stored in barrier material PET/Al/PE under atmospheric conditions and modified atmosphere and 17–22 °C, dry lemon slices stored in barrier pouches under atmospheric conditions or N_2_ modified atmosphere and (25 ± 3 °C) and dried pomegranate arils stored in high-barrier pouches under atmospheric conditions at 38 ±1 °C [10,12,51,61]. TP was shown to be the main phytochemical contributing to the antioxidant activity in fruits and their linear correlation to antioxidant activity was shown in dry papaya samples [51,62,63]. These results are consistent with the findings of this study where a decrease in total phenol content was followed by a decrease in the antioxidant activity of samples (FRAP and ABTS tests). The different trend of DPPH values could be explained by the diversity of FRAP, ABTS, and DPPH assays since they provide three distinct tests for assessing antioxidant capacity based on various compounds that display potential to scavenge or reduce. The main goal for using multiple antioxidant assays was to obtain a better overview of the antioxidant profile. The decrease in TP content, as explained by the authors, was probably due to some of the following factors, or their combination: activity of oxidative enzymes including polyphenol oxidase or chemical oxidation, storage temperature, pH, exposure to oxygen and light or increase in moisture content [51,64]. The decrease in TP in this study (18.4–25.7%) was considerably lower than in dry papaya samples (40%), dry lemon slices (60–70%), and dry apricot samples (60%) during the same storage period, most likely due to the lower temperature of storage (23 ± 2 °C), the absence of oxygen and light [65], and the preservation of low moisture content in the samples.

Total flavonoid content was found to slowly decrease during storage. The main decrease was recorded after 15 days of storage. Literature data show that dry fruit packaging under a N_2_ atmosphere and room conditions in high-barrier packaging materials can keep the flavonoid content constant during storage or with a lower decrease [10]. Further minimization of flavonoid losses during storage might be achieved by combining low-temperature storage with N_2_-modified packaging [66].

Drying, as a preservation technology, increases fruit shelf life, retarding the growth of spoilage microorganisms. It was reported that optimum a_w_ for microorganism growth is in the range of 0.99–0.98, while the majority of microorganisms are unable to grow when a_w_  <  0.90. Water activity in this sense could be considered an important indicator of fruit shelf life, considering that it is of vital importance for potential microbial growth [67]. Water activity of the tested samples at the beginning of the storage period was 0.364 ± 0.005 and until the end of 105 days of storage, it remained around 0.400 in both tested packaging systems without considerable difference between them (*p* > 0.05).

Although foods with a_w_ below 0.9 are regarded as unfavorable environments for microbial growth, there is literature confirmation that the lowest a_w_ at which growth of bacteria has been disclosed was 0.75, but fungal growth was reported at a_w_ levels near 0.6 [67,68]. A high reduction in a_w_ between 0.2 and 0.4, such as in this paper, may have an antagonistic effect with heat, leading to higher resistance of both spores and vegetative cells to thermal stress [69,70,71]. Also, the survival of microorganisms in dry fruits can be influenced by the rate of initial contamination during harvesting, storage, production (handling, slicing) and transportation, where the only step reducing microbial counts might be the drying step. Introduced microorganisms (also pathogens) can survive the drying process and vegetative cells and spores can preserve viability for weeks during storage until consumption. This is especially important for dry food that is being consumed without any further treatment, like dry fruits [68,69,72]. This is where multiple stresses might be of great benefit, like the combined action of low a_w_ and low-oxygen atmosphere during storage, as in this study. While Salmonella and Escherichia coli have been found in dried food products [68,69,72,73], in this research, no presence of pathogenic bacteria was detected throughout the storage period in both packaging systems.

Similar to the report of Ntuli et al. [61] for commercially produced dry apricots, in the present research (Table 3), only the presence of molds, yeasts and aerobic mesophilic bacteria was detected. According to microbiological guideline criteria used for ready-to-eat foods, ready-to-eat foods with standard plate counts lower than 103 are considered satisfactory, carrying no food safety concerns [74]. Yeast number was reduced after drying and after 15 days of storage under a modified atmosphere, yeasts were not detected anymore, and this was the case throughout the storage in both PuOC/wax and OPPmat/OPPmet pouches. Mold and aerobic mesophilic bacteria counts were also decreased through the storage in both packaging systems and this decrease was more pronounced in PuOC/wax pouches, especially for molds (*p* < 0.05). It could be said, according to the results of the microbiological examination, that a safe product was obtained throughout the packing and storage in both analyzed packaging systems.

## 4. Conclusions

In this study, new packaging material was obtained based on composite biopolymer hydrogel-based material derived from pumpkin oil cake, an edible oil by-product, coated with paraffin wax. Characterization of the new packaging material revealed mechanical and water sensitivity properties that resemble composite biopolymer film base properties, to a certain extent. However, applying a thin layer of paraffin wax to the composite biopolymer film notably decreased both the moisture content and the rate of water vapor transmission. The tested material was used to form pouches intended for sensitive low-water content foods under modified atmosphere conditions. As a model food, dried apricot slices were packed under a modified atmosphere. Headspace gas composition during 3,5 months of storage at room conditions confirmed the promising performance of the new material, resulting in a minimally changed modified atmosphere. For dried apricot slice quality parameters, the following observations were made: no notable detected in the moisture content of apricots, compared to the initial moisture content, a very high level of rehydration power, unchanged sensorial properties, minimal decrease in total phenols content and antioxidant activity and low microbial growth, indicating a safe product. According to these results, it can be concluded that the functionality of the new PuOC-wax packaging material for packing dry foods under a modified atmosphere was proven.

## Figures and Tables

**Table 1:** Properties of PuOC, PuOC/wax and OPPmat/B/adh/OPPmet packaging materials.

Packaging Material	Film Thickness (mm)	Tensile Strength (N/15 mm)	Elongation at Break (%)	Swelling Degree (%)	Moisture Content (%)	Total Soluble Matter (%)	Water Vapor Transmission Rate (g/m^2^ 24 h)
PuOC *	0.120 ± 0.020	7.5 ± 1.1	108.60 ± 12.28	235.01 ± 41.79	20.17 ± 1.06	35.14 ± 1.60	347.52 ± 19.96
PuOC/wax	0.141 ± 0.032	13.3 ± 0.7	76.62 ± 0.50	234.04 ± 33.12	9.92 ± 1.12	41.86 ± 3.45	56.98 ± 7.42
OPPmat/B/adh/OPPmet	0.047	Parallel 85.0 ± 2.7 Normal 159.6 ± 6.9	Parallel 278.63 ± 16.90 Normal 79.45 ± 6.47	-	-	-	<1

* Literature data, given for comparison only [17].

**Table 2:** Parameters of dried apricot slice quality during 105 days of storage in PuOC and OPPmat/B/adh/OPPmet-based pouches.

	Days of Storage							
	0		15		45		105	
Packaging material	PuOC/ wax	OPPmat/OPPmet	PuOC/ wax	OPPmat/OPPmet	PuOC/wax	OPPmat/OPPmet	PuOC/wax	OPPmat/OPPmet
TP (mg GAE/100 g)	341.03 ^a^ ± 2.81		302.67 ^b^ ± 3.37	271.16 ^c^ ± 3.29	276.15 ^c^ ± 4.78	320.13 ^d^ ± 1.95	278.33 ^c^ ± 3.37	253.38 ^e^ ± 3.54
TF (mg CE/100 g)	150.57 ^a^ ± 8.55		85.83 ^b^ ± 2.15	92.00 ^bc^ ± 2.49	92.99 ^bc^ ± 5.46	100.36 ^c^ ± 2.41	100.165 ^c^ ± 1.58	95.53 ^b^ ± 1.19
MC (%)	11.44 ^a^ ± 0.52		10.51 ^ab^ ± 0.29	10.39 ^ab^ ± 0.23	10.18 ^b^ ± 0.60	10.76 ^ab^ ± 0.43	10.45 ^ab^ ± 0.27	10.60 ^ab^ ± 0.53
a_w_	0.364 ^a^ ± 0.005		0.412 ^b^ ± 0.013	0.331 ^c^ ± 0.006	0.377 ^ad^ ± 0.017	0.378 ^ad^ ± 0.018	0.400 ^bd^ ± 0.010	0.396 ^ab^ ± 0.008
RP (%)	70 ^a^ ± 0.70		75 ^b^ ± 0.58	65 ^c^ ± 0.62	70 ^a^ ± 0.44	65 ^c^ ± 0.32	70 ^a^ ± 0.75	65 ^c^ ± 0.39
TSS	20 ^a^ ± 0.0		19.8 ^ab^ ± 0.4	18.9 ^bc^ ± 0.7	19.2 ^abc^ ± 0.4	19.1 ^bc^ ± 0.5	18.9 ^c^ ± 0.4	18.7 ^c^ ± 0.3
DPPH (mg Trolox/g)	11.93 ^a^ ±0.392		10.48 ^b^ ± 0.341	10.70 ^b^ ± 0.233	11.23 ^ab^ ± 0.281	12.79 ^c^ ± 0.281	11.26 ^ab^ ± 0.129	15.73 ^d^ ± 0.258
FRAP (mg Fe^2+^/g)	4.67 ^a^ ± 0.054		2.39 ^b^ ± 0.037	2.59 ^c^ ± 0.074	4.10 ^d^ ± 0.062	2.77 ^e^ ± 0.037	2.66 ^ce^ ± 0.027	3.04 ^f^ ± 0.053
ABTS (mg Trolox/g)	2.44 ^a^ ± 0.364		0.738 ^bc^ ± 0.429	0.321 ^b^ ± 0.252	0.698 ^bc^ ± 0.137	0.817 ^bc^ ± 0.300	0.103 ^b^ ± 0.038	1.21 ^c^ ± 0.238

^a,b,c,d,e,f^ Different letters denote significantly different means with 95% probability (*p* < 0.05) within rows.

**Table 3:** Microbiological profile of dried apricot slice quality during 105 days of storage in PuOC and OPPmat/B/adh/OPPmet-based pouches.

		Days of Storage							
	Before Drying	0		15		45		105	
Packaging material	-	PuOC/ wax	OPPmat/ OPPmet	PuOC/ wax	OPPmat/OPPmet	PuOC/ wax	OPPmat/ OPPmet	PuOC/ wax	OPPmat/ OPPmet
Total aerobic mesophilic bacteria count—TBC (CFU/g)	450 ^a^ ± 50	360 ^a^ ± 40		400 ^a^ ± 100	500 ^a^ ± 100	60 ^b^ ± 10	120 ^b^ ± 30	50 ^b^ ± 20	100 ^b^ ± 0
Total yeast count (CFU/g)	150 ^a^ ± 20	70 ^b^ ± 10		<10	<10	<10	<10	<10	<10
Total mold count (CFU/g)	360 ^a^ ± 40	440 ^a^ ± 60		60 ^bd^ ± 10	220 ^c^ ± 20	20 ^b^ ± 10	120 ^d^ ± 10	<10	70 ^bd^ ± 20
Total mesophilic sporogenic count (CFU/g)	<10	<10		<10	<10	<10	<10	<10	<10
Total *Enterobacteriaceae* count (CFU/g)	<10	<10		<10	<10	<10	<10	<10	<10
*E. coli* (CFU/g)	<10	<10		<10	<10	<10	<10	<10	<10
*Salmonella* spp. (CFU/g)	n.d.	n.d.		n.d.	n.d.	n.d.	n.d.	n.d.	n.d.
*L. monocytogenes* (CFU/g)	n.d.	n.d.		n.d.	n.d.	n.d.	n.d.	n.d.	n.d.

^a,b,c,d^ Different letters denote significantly different means with 95% probability (*p* < 0.05) within rows.

## Data Availability

The original contributions presented in this study are included in the article. Further inquiries can be directed to the corresponding authors.
